# A hypothesis-generating study to evaluate platelet activity in diabetics with chronic kidney disease

**DOI:** 10.1186/1477-9560-3-3

**Published:** 2005-03-29

**Authors:** Donald F Brophy, rika J Martin, Todd WB Gehr, Marcus E Carr

**Affiliations:** 1 Department of Pharmacy Practice, Virginia Commonwealth University/Medical College of Virginia Campus (VCU/MCV), Richmond, VA, USA; 2 Department of Internal Medicine, VCU/MCV, Richmond, VA, USA; 3 Coagulation Special Studies Laboratory, VCU/MCV, Richmond, VA, USA; 4 Department of Pathology, VCU/MCV, Richmond, VA, USA

**Keywords:** diabetes, thrombin generation, platelet function, renal dysfunction

## Abstract

**Background:**

It is well described that diabetes mellitus is a hypercoagulable state. It is also known that patients with renal dysfunction have impaired platelet aggregation and function. It is not well described how renal dysfunction affects the hypercoagulability associated with diabetes. This post-hoc sub-group analysis compares platelet function, clot structure and thrombin generation time at baseline, and following enoxaparin exposure in three groups of subjects.

**Methods:**

30 total subjects were evaluated in the three groups: Group I: normal controls (n = 10), Group II: subjects with renal dysfunction but without diabetes (n = 13), and Group III: subjects with concomitant diabetes and renal dysfunction (n = 7). For each subject, platelet contractile force (PCF), clot elastic modulus (CEM) and thrombin generation time (TGT) were simultaneously measured in whole blood at baseline, and following increasing enoxaparin antifactor Xa activity exposure. The group means for each parameter were determined and compared using one-way analysis of variance, with post-hoc Tukey-Kramer test.

**Results:**

At baseline, subjects in Group III (diabetics with concomitant renal dysfunction) display significantly enhanced platelet activity, as measured by PCF (p = 0.003) and CEM (p = 0.03), relative to the non-diabetic Groups I and II. Subjects in Group II (renal dysfunction without diabetes) had significantly prolonged TGT values relative to controls when the antifactor Xa activity concentration reached 0.5 (p = 0.007), 1.0 (p = 0.005) and 3.0 IU/mL (p < 0.0001), respectively. There were no differences between Group II and Group III with respect to TGT at these antifactor Xa activity concentrations. When the antifactor Xa activity concentration reached 3.0 IU/mL, Groups II and III formed significantly less rigid blood clots (CEM p = 0.003) and also trended toward reduced PCF (p = 0.06) relative to Group I.

**Conclusion:**

This hypothesis-generating sub-group analysis suggests that at baseline, patients with concomitant diabetes and renal dysfunction have significantly enhanced platelet activity (PCF), and form more rigid blood clots (CEM) compared to controls and subjects with renal dysfunction but no diabetes. This may suggest that the presence of renal dysfunction does not ameliorate the hypercoagulable state associated with diabetes. Secondly, it appears that subjects with renal dysfunction but without diabetes have an enhanced response to enoxaparin relative to controls.

## 

Diabetes mellitus is a hypercoagulable state [1]. It has been demonstrated that patients with diabetes have abnormally elevated markers of coagulation activation [2-12], as well as increased risk of mortality from thrombosis [13]. Indeed, virtually all Type II diabetics will die a thrombotic death, whether its origin be cardiac, cerebrovascular or peripheral vascular [14].

What is less clear is how the presence of renal dysfunction affects the hypercoagulable state associated with diabetes. This is an important question; given the fact that renal dysfunction is so prevalent in patients with diabetes [15]. There is clinical and laboratory evidence that uremia associated with renal dysfunction leads to platelet dysfunction and altered hemostasis. [16-20]. However, we are unaware of any reports that suggest thrombotic risk decreases in diabetics who develop renal dysfunction.

Our laboratory has developed a novel whole blood monitoring assay that determines thrombin generation, platelet function and clot structure on a single sample of patient blood. This assay provides a global assessment of clotting function by reporting the markers platelet contractile force (PCF), clot elastic modulus (CEM) and the onset of thrombin generation as measured by thrombin generation time (TGT). PCF is the force produced by platelets during clot retraction, and is a novel measure of platelet function during clotting; CEM reflects the structural integrity of the blood clot; and TGT is the speed at which thrombin is produced in whole blood [21]. Changes in these parameters provide a detailed characterization of the hemostatic effect of various disease states [22,23], antithrombotic and anticoagulant drugs [21,24,25], and hemostatic agents [26] on platelet function and thrombin generation.

We report here findings of a hypothesis-generating sub-group analysis that illustrate the effects of diabetes and renal dysfunction on platelet function and thrombin generation time. In addition, we examined the potential impacts of these disease states on the response to the commonly used low-molecular weight heparin enoxaparin.

## Patients and Methods

This was a hypothesis-generating sub-group analysis of a prospective, open-label, *ex vivo *study of enoxaparin in patients with and without renal dysfunction [27]. The Virginia Commonwealth University (VCU) Institutional Review Board approved this study prior to subject enrollment, and this study was conducted in compliance with the Declaration of Helsinki. In this study, a total of 30 subjects were evaluated: 10 healthy controls; 13 subjects with renal dysfunction but without diabetes; and seven patients with concomitant diabetes and renal dysfunction (6 CKD and 1 ESRD).

All subjects were anticoagulant naïve, non-thrombosed, and otherwise healthy. Subjects were admitted into this study if they were > 18 years of age and provided written informed consent. In addition, subjects with ESRD must have received maintenance hemodialysis for at least 3 months through an arterio-venous fistula or graft. Subjects were excluded from this investigation if they had any of the following: active bleeding or thrombotic disorder; pregnancy; recent trauma or surgery (< 7 days); cirrhosis or other liver abnormality; hematocrit < 30%; active cancer; had received a blood transfusion within 1 week of study enrollment; thrombocytopenia (platelets < 100,000/mL); documented history of antithrombin III, protein C or protein S deficiency; concurrent use of anticoagulant or antiplatelet drug therapy. All subjects who provided written informed consent and who met the above criteria underwent screening evaluation, which consisted of a routine physical exam and laboratory evaluation (basic metabolic panel, complete blood count (CBC), international normalized ratio (INR), activated partial thromboplastin time (aPTT), prothrombin time (PT)).

### Study Procedures

For each subject, blood was collected via aseptic venipuncture into four evacuated tubes containing 3.2% sodium citrate. The blood was pooled and then aliquotted into five separate samples, which were spiked with increasing enoxaparin antifactor Xa activity concentrations. Final enoxaparin antifactor Xa concentrations in the respective aliquots were 0.0, 0.25, 0.50, 1.0, and 3.0 IU/mL. For each aliquotted blood sample, analysis was performed to determine the corresponding TGT, PCF and CEM at each antifactor Xa activity concentration.

### Specimen Processing and Analysis

Baseline chemistries (basic metabolic profile, CBC, PT, aPTT, INR) and antifactor Xa concentrations were processed and analyzed at the VCU Health System Department of Pathology. Plasma enoxaparin antifactor Xa activity was measured using a validated, commercially available chromogenic method (STA^® ^heparin colorimetric analyzer, Diagnostica Stago, Parsippany, NJ, USA), and the results expressed as IU/mL. The lower limit of antifactor Xa detection for this assay was 0.05 IU/mL; the coefficient of variation (CV) was ± 3%. Whole blood samples were analyzed for TGT, PCF and CEM at the VCU Coagulation Special Studies Laboratory. The TGT, PCF and CEM for each aliquotted whole blood sample were simultaneously measured using the Hemodyne Hemostasis Analysis System™ (Hemodyne, Inc., Richmond, VA, USA) using a previously published, validated method [27]. All samples were run in duplicate. The CV for this assay was ± 7%.

### Statistical Analysis

All statistical analyses were performed using JMP statistical software version 5.1 (SAS Institute, Cary, NC, USA). The data were presented based on subject group assignment, as Group I: Control; Group II: subjects with renal dysfunction but without diabetes; and Group III: subjects with concomitant diabetes and renal dysfunction. Descriptive statistics characterized the group demographic data One-way analysis of variance (ANOVA) assessed for intergroup differences in demographic data, and mean PCF, CEM and TGT at baseline and at each spiked enoxaparin antifactor Xa activity concentration for each of the three groups. If intergroup differences were found to be statistically significant, a post-hoc Tukey-Kramer test was used to differentiate which groups were statistically different. The level of significance for all statistical tests was p < 0.05.

## Results

Thirty subjects completed this study as described above. There were no adverse reactions or dropouts from the study. Table 1 details the subject demographics. Other than differences in the racial makeup of the groups, the groups were similar with respect to age, sex, weight and presence of coronary artery disease (CAD).

**Table 1:** Baseline Subject Demographics

	**Group I**	**Group II**	**Group III**	
**Parameter**	**Controls (n = 10)**	**Renal Dysfunction (n = 13)**	**Diabetes and Renal Dysfunction (n = 7)**	**P-value**
**Age **[mean (S.D.)]	40.5 (9.6)	46.5 (15.0)	45.1 (11.1)	NS
**Race **[number (%)]				
Caucasian	5 (50)	1 (8)	0	
Black	3 (30)	12 (92)	6 (86)	0.02^§^
Other	2 (20)	0	1 (14)	
**Sex **[number (%)]				
Male	5 (50)	7 (54)	3 (43)	NS
Female	5 (50)	6 (46)	4 (57)	
**Weight**				
[mean kg (S.D.)]	75.0 (13.4)	83.9 (23.1)	86.0 (25.0)	NS
**CAD **[number (%)]				
Yes	1 (10)	2 (15)	3 (43)	NS
No	9 (90)	11 (85)	4 (57)	

NS – Not significant

§ Chi-square test for group homogeneity significantly different

### Baseline Platelet Function, Clot Structure and Thrombin Generation Time

The top panel of Table 2 details the baseline PCF, CEM and TGT in Groups I, II and III. Group III (concomitant diabetes and renal dysfunction) exhibited significantly greater platelet activity, as measured by PCF (p = 0.003) and CEM (p = 0.03), relative to the non-diabetic groups I and II. The reported TGT was not significantly different between groups at baseline.

**Table 2:** Mean (S.D.) Platelet Contractile Force, Clot Elastic Modulus and Thrombin Generation Time at Baseline and Following Increasing Enoxaparin Antifactor Xa Activity

	**Group I**	**Group II**	**Group III**	
**Antifactor Xa Activity**	**Controls (n = 10)**	**Renal Dysfunction (n = 13)**	**Diabetes and Renal Dysfunction (n = 7)**	**P-value**
**Baseline**				
PCF^†^	8.5 (1.4)	9.8 (1.5)	12.0 (2.9)*	0.003
CEM^‡^	23.2 (5.4)	29.0 (9.5)	37.3 (15.5)*	0.03
TGT^§^	222.0 (60.3)	235.4 (53.2)	270.0 (52.0)	NS
				
**0.25 IU/mL**				
PCF	7.0 (1.8)	7.5 (1.6)	8.8 (2.9)	NS
CEM	21.1 (4.1)	22.6 (4.1)	24.6 (7.1)	NS
TGT	273.0 (68.5)	327.7 (70.8)	325.7 (76.3)	NS
				
**0.50 IU/mL**				
PCF	5.5 (1.9)	5.6 (1.8)	6.3 (2.4)	NS
CEM	17.2 (5.1)	19.1 (4.4)	19.4 (5.0)	NS
TGT	318.0 (68.1)	440.0 (97.5)*	420.0 (86.6)	0.007
				
**1.0 IU/mL**				
PCF	3.6 (1.7)	3.0 (1.7)	4.0 (2.1)	NS
CEM	11.9 (4.5)	10.0 (4.6)	12.9 (6.1)	NS
TGT	423.0 (128.4)	588.5 (96.0)*	552.9 (114.7)	0.005
				
**3.0 IU/mL**				
PCF	1.5 (1.3)	0.6 (0.2)	1.0 (0.9)	0.06
CEM	4.8 (4.4)*	0.8 (0.8)	0.9 (0.8)	0.003
TGT	780.0 (317.5)	1200.0 (0)*	1200.0 (0)*	< 0.0001

^† ^PCF – Kdynes, ^‡ ^CEM – Kdynes/cm^2^,^§^TGT – seconds

NS – not significant

* Statistically significant

### Comparative Platelet Function, Clot Structure and Thrombin Generation Times Following Increasing Enoxaparin Antifactor Xa Activity Concentrations

As the spiked *ex vivo *enoxaparin antifactor Xa activity concentration increased, there were corresponding changes in PCF, CEM and TGT (Table 2). As expected, there was an inverse relationship between increasing enoxaparin antifactor Xa activity and platelet function (i.e., PCF) and clot structure (i.e., CEM) in all three groups. Conversely, there was a direct relationship between TGT prolongation and increasing enoxaparin antifactor Xa activity in the groups.

The PCF and CEM were not statistically different between groups at antifactor Xa activity concentrations of 0.25, 0.5 or 1.0 IU/mL. However, when the antifactor Xa activity concentration reached 3.0 IU/mL, Group I (controls) had significantly higher CEM (p = 0.003) relative to the two groups with renal dysfunction. There was also a trend of greater PCF in Group I relative to the other groups.

The TGT was significantly prolonged in Group II (renal dysfunction without diabetes) relative to Group I (controls) at enoxaparin antifactor Xa activity concentrations of 0.5 (p = 0.07), 1.0 (p = 0.005) and 3.0 IU/mL (p < 0.0001), respectively. There were no statistical differences between Groups II and III (concomitant diabetes and renal dysfunction) at these antifactor Xa activity concentrations.

## Discussion

This was a hypothesis-generating sub-group analysis performed to assess the pro-coagulant effect of diabetes on the platelet function in patients with CKD. To our knowledge, this is the first study to demonstrate that at baseline, diabetic patients with renal dysfunction have elevated platelet activity. Our review of the literature fails to identify prior studies of platelet function specifically performed in diabetics with renal dysfunction. Given the apparently divergent effects of hyperglycemia and uremia on platelet function, one might hypothesize that depressed platelet function due to uremia may be partially or completely reversed by the enhanced effects of diabetes. At baseline, this appears to be the case. The presence of diabetes increases platelet forces, vis-à-vis PCF and results in greater clot strength, vis-à-vis CEM, compared to the other non-diabetic groups I and II. However, despite the increased platelet activity in diabetics with renal dysfunction noted at baseline, there were no differences in TGT between groups.

Previous studies of platelet function in diabetes or uremia have primarily involved measurement of platelet aggregation. Enhanced platelet aggregation has been demonstrated with diabetes [3,29,30] and decreased platelet aggregation has been identified in uremia [31-35]. Previous studies of PCF noted enhanced function in diabetes [23], but normal values in chronic uremia [36]. These studies are consistent with the current findings. The divergence between aggregation results and PCF findings centers around the fact that one assay (platelet aggregation) is performed with minimal platelet activation and the other (PCF) under conditions of maximal platelet activation (i.e., platelets in the presence of thrombin). Thrombin is such a profound agonist of platelet function that subtle influences noted by platelet aggregation studies are simply overwhelmed. An example of this effect can be seen with the effects of IIb/IIIa blockade on platelet aggregation and PCF. The concentrations of IIb/IIIa blocking agents (e.g., abciximab, tirofiban) required to block PCF production are an order of magnitude higher than those required to suppress platelet aggregation [24].

The data from this analysis, although limited by the relatively small sample size, suggests that there is a "counter-balancing" effect of renal dysfunction and diabetes with respect to platelet function and thrombin generation time. This is borne out in Table 2. For example, subjects in Group II (renal dysfunction but without diabetes) have the greatest prolongations in TGT, compared to the other groups, following increasing antifactor Xa activity. However, Group III (renal dysfunction with diabetes) subjects tend to have a blunted TGT prolongation, though not significantly different compared to Group II. One could argue that given a larger sample size, potential differences may be detected between Groups II and III. Our findings should at least caution against assuming the presence of an "auto" anticoagulant or "auto" antiplatelet effect of uremia in patients with concomitant diabetes.

There are important limitations to these findings. First, this study was a sub-group analysis not designed *a priori *to detect these differences. Indeed, these differences were detected on data analysis and thought to be important hypothesis-generating data. Secondly, the groups presented were quite heterogeneous, both in terms of numbers and underlying disease states. That is, there were control patients with neither diabetes nor renal dysfunction, there were patients with solely renal dysfunction but no diabetes, and there were patients with concomitant diabetes and renal dysfunction. A more appropriate way to study the effects of diabetes and renal dysfunction on platelet activity and thrombin generation time is prospectively using four study groups: 1) non-diabetics without CKD; 2) non-diabetics with CKD; 3) diabetics without CKD; and 4) diabetics with CKD. This study design would more appropriately describe the effect of diabetes on uremic platelets. Moreover, this would allow for greater statistically analysis using a two-way ANOVA test, that could detect an interaction between the concomitant disease states on altered platelet function. Nevertheless, these data are compelling enough to develop a hypothesis for further study.

## Conclusion

In conclusion, we report hypothesis-generating findings that suggest that at baseline subjects with concomitant diabetes and renal dysfunction have increased platelet activity relative to healthy controls and patients with renal dysfunction but no concomitant diabetes. This may suggest that the presence of uremia does not completely ameliorate the hypercoagulable effect of diabetes. Secondly, it appears that subjects with renal dysfunction but without concomitant diabetes have an enhanced response to enoxaparin compared to the controls, as measured by TGT. Further large-scale studies are warranted to further characterize the effects and interactions of diabetes and renal dysfunction on platelet function.

## Abbreviations

PCF, platelet contractile force; CEM, clot elastic modulus, TGT, thrombin generation time, CKD, chronic kidney disease; ESRD, end-stage renal disease; GFR, glomerular filtration rate; VCU, Virginia Commonwealth University; LMWH, low-molecular weight heparin; CBC, complete blood count; INR, international normalized ratio; aPTT, activated partial thromboplastin time; PT, prothrombin time, CAD, coronary artery disease; ANOVA, analysis of variance.

## Competing Interests

There are no financial or non-financial competing interests related to this manuscript.

## Authors' Contributions

DFB was the principal investigator for the study and was responsible for all aspects of its conduct.

EJM was the laboratory technologist who performed all analytical assays.

TWBG was responsible for recruitment of dialysis subjects and study design.

MEC was responsible for overseeing all analytical activities, as well as study design and data analysis.
